# Clonal diversity, virulence genes content and subclone status of *Escherichia coli* sequence type 131: comparative analysis of *E. coli* ST131 and non-ST131 isolates from Iran

**DOI:** 10.1186/s12866-019-1493-8

**Published:** 2019-05-30

**Authors:** Zoya Hojabri, Narges Darabi, Maedeh Arab, Fereshteh Saffari, Omid Pajand

**Affiliations:** 1 Microbiology Department, Faculty of Medicine, Semnan University of Medical Sciences, Semnan, Iran; 2 Student Research Committee, Faculty of Medicine, Semnan University of Medical Sciences, Semnan, Iran; 3 Microbiology Department, Faculty of medicine, Kerman University of Medical Sciences, Kerman, Iran; 4 Social Determinants of Health Research Center, Semnan University of Medical Sciences, Semnan, Iran

**Keywords:** ST131, Carbapenem, NDM, OXA-48, Avian, Virulence genes, Antibiotic resistance

## Abstract

**Background:**

The *Escherichia coli* sequence type 131 (ST131) is a well established clone causing significant extraintestinal infections worldwide. However, no studies have been reported the phenotypic and molecular traits of ST131 isolates in comparison to other clones of *E. coli* from Iran. So, we determined the differences between 69 ST131 strains collected during a one year surveillance study and 84 non-ST131 isolates, including 56 clinical fluoroquinolone resistant and 28 broiler colibacillosis isolates in terms of clonality and genetic background.

**Results:**

ST131 isolates were associated with phylogroup B2 (68 out of 69 isolates, 98.4%), while clinical non-ST131 and fluoroquinolone resistant broiler isolates mainly belonged to phylogroup A. The highest virulence score was observed in ST131 clone, while they showed less diversity in virulence profiles than other clinical isolates. Almost all of the ST131 isolates (95.6%) were ExPEC and had the highest virulence scores, but their resistance scores were less than clinical non-ST131 isolates. Broiler isolates showed higher prevalence of ExPEC-associated virulence genes and CTX-M-G1/G9 resistance determinants as compared to clinical non-ST131 isolates. While *bla*_OXA-48/NDM_ carbapenemases were mostly found in ST131 clone, resistance rate against ertapenem was higher among clinical non-ST131 strains. According to ERIC-based fingerprinting, the ST131 strains were more genetically similar, followed by non-ST131 and broiler isolates.

**Conclusions:**

ST131 isolates possess the ability to make a balance between clonality and extent of resistance/virulence genes content, so this phenomenon gives a fitness advantage over other *E. coli* clones. The broilers *E. coli* population poses a potential zoonotic risk which could be transmitted to the community through the food chain. A number of factors are involved in the dissemination of and infections due to ST131 clone.

## Background

Extraintestinal infections caused by *Escherichia coli*, including urinary tract infections, bloodstream infections and neonatal meningitis are common, costly and are major causes of morbidity and mortality. Emerging antimicrobial resistance complicates the management of these infections [1].

*E. coli* sequence type 131 (ST131) is defined by multilocus sequence typing (MLST) technique and is characterized by resistance to first line antibiotics such as fluoroquinolone and extended spectrum cephalosporins (ESCs). Two major subclones which were identified within the ST131 population are *H*30 and *H*30Rx. Except for rare strains, almost all *H*30 isolates are fluoroquinolone resistant, while *H*30Rx subclone has been associated with Extended Spectrum β-lactamases (ESBLs), namely CTX-M-15 [2, 3].

Fluoroquinolones are widely used in farm animals, mainly in broilers [4, 5] and fluoroquinolone resistant *E. coli* strains are frequently isolated from healthy and diseased birds [4]. It has been hypothesized that avian *E. coli* isolates are reservoirs of virulence and resistance determinants, and strains harboring these elements may be able to cause foodborne diseases in humans. Characterization of overlapping traits between avian pathogenic *E. coli* (APEC) and urinary pathogenic *E. coli* (UPEC), such as O-serogroups, virulence markers and assignments to phylogroups, encouraged this hypothesis and subsequent researches comparing APEC and human *E. coli* isolates including UPEC, neonatal meningitis *E. coli* (NMEC) and *E. coli* causing septicemia showed common molecular traits between human and avian strains [4, 6, 7]. While the presence of ST131 clone among *E. coli* isolates contaminating raw chicken meat wasn’t confirmed in the studies from Spain and Italy [8, 9], another survey from Spain found this specific clone in 7% of retail chicken samples [10]. Vincent et al. also had identified *E. coli* ST131 from retail chicken samples in Canada but at a significant lower prevalence (0.4%) than in Spain [11].

The presented reasons for the success of the ST131 pandemic clone are the acquisition of antimicrobial resistance and additional virulence factors as well as its predominance in the human gut. There are some reports which suggest that epidemiological differences exist between CTX-M-producing strains of ST131 and non-ST131 clones [12]. Our previous study from Iran reported important data on clinical *E. coli* ST131 isolates [13]. However, a comparative work describing fluoroquinolone resistant/ESBL producing ST131 *E. coli* and other non-ST131 isolates or isolates from avian sources has not been reported so far from Iran. Therefore, the objectives of this study were to compare the clonality, virulence characteristics and resistance genotypes of ST131 isolates with that of human non-ST131 and broiler colibacillosis strains in order to determine whether these genotypes were shared or not between these groups of isolates.

## Results

### Human-sourced *E. coli* isolates

Sixty-nine strains out of 338 collected human *E. coli* isolates belonged to ST131 clonal group. The O25b/O16 serogroups, *H*30/*H*30Rx subclones and virotypes of 63 isolates have been previously described [13]. The remaining six newly identified ST131 isolates belonged to phylogroup B2, O25b subgroup, virotype C and *H*30 subclone. Of the remaining 269 *E. coli* isolates, 56 fluoroquinolone resistant isolates were randomly selected. Thus, a total of 125 unique human *E. coli* isolates (isolated from 125 admitted patients) were subjected to the thorough investigation described below. Among the ST131 isolates, 39 (56.5%) were from female patients with a mean age of 65.49 years and 30 (43.5%) were from male patients, with a mean age of 73 years, whereas among non-ST131 isolates 39 (69.6%) were from female patients and 17 (30.4%) were from male patients with mean ages of 66.6 and 64.8 years, respectively. Overall, specimen types for ST131 and clinical non-ST131 isolates included blood (0 and 1.8%), wound (5.8% and 1.8%), respiratory (11.6% and 12.5%) and urine (82.6% and 83.9%). The prevalence of ST131 and clinical non-ST131 isolates were higher among women, however, sex distribution did not vary significantly between two groups of clinical isolates.

### Prevalence of ESBL-producing *E .coli* and antimicrobial susceptibility

Fluoroquinolone resistance was detected among 63 ST131 (all belonged to subgroup O25b) and 18 (64.2%) broiler isolates. The highest frequency of resistance was obtained against cefotaxime (95.7%), cefotaxime (100%) and trimethoprim/sulfamethoxazole (71.4%) among ST131, non-ST131 and broiler isolates, respectively. Overall, except for carbapenems, a high proportion of ESBL producing isolates were non-susceptible to antibiotics tested and were categorized as MDR (98.4%, 121/123, *P*: 0.001).

The resistance rates against studied antibiotics among non-ST131 isolates were higher than ST131 and the differences were significant for trimethoprim/sulfamethoxazole, amoxicillin/clavulanate, piperacillin-tazobactam, ampicillin/sulbactam, fluoroquinolones and tobramycin (Table 1). The highest resistance score was obtained among non-ST-131 (median: 9, IQR: 3) as compared with ST131 (median: 8, IQR: 4, *P*: 0.01) and broiler isolates (median: 2, IQR: 2, *P*: 0.001).

**Table 1:** Antibiotic resistance patterns and molecular determination of resistance genes in studied isolates

ST131 (*n* = 69)				Non-ST131 (*n* = 84)			
antibiotics	O25b-ST131 *n* = 63	O16-ST131 *n* = 6	Total ST131	Human (*n* = 56)	broiler (*n* = 28)	*P* value	
						*ST131* vs. *non-ST131*	*ST131* vs *broilers*
Antibiotic resistance rates, n(%)							
Imipenem	1 (1.6)	1 (16.7)	2 (2.9)	0	0		
Meropenem	1 (1.6)	0	1 (1.4)	1 (1.8)	0		
Ertapenem	8 (12.7)	1 (16.7)	9 (13)	12 (21.4)	0		0.05
Ceftazidime	49 (77.8)	5 (83.3)	54 (78.3)	48 (85.7)	1 (3.6)		0.001
Cefepime	37 (58.7)	4 (66.7)	41 (59.4)	37 (66.1)	1 (3.6)		0.001
Cefotaxime	61 (96.8)	5 (83.3)	66 (95,7)	56 (100)	1 (3.6)		0.001
Ciprofloxacin/levofloxacin	63 (100)	0	63 (91.3)	56 (100)	18 (64.3)	0.03	0.002
Piperacillin/tazobactam	10 (15.9)	2 (33.3)	12 (17.4)	24 (42.8)	1 (3.6)	0.003	
Ampicillin/sulbactam	27 (43.5)	5 (83.3)	32 (46.4)	39 (69.6)	0	0.01	0.001
Amoxicillin/clavulanate	36 (57.1)	5 (83.3)	41 (59.4)	44 (78.6)	4 (14.3)	0.03	0.001
Aztreonam	59 (93.7)	5 (83.3)	64 (92.8)	52 (93)	1 (3.6)		0.001
Trimethoprim/sulfameth	44 (69.8)	5 (83.3)	49 (71)	48 (85.7)	20 (71.4)	0.05	
Tobramycin	32 (50.8)	1 (16.7)	33 (47.8)	38 (67.8)	3 (10.7)	0.03	0.001
Amikacin	7 (11.1)	0	7 (10.1)	8 (14.3)	0		
Gentamicin	29 (46)	2 (33.3)	31 (44.9)	33 (59)	1 (3.6)		0.001
Resistance score (median)	8	8	8	9	2	0.01	0.001
ESBL phenotype	61 (96.8)	5 (83.3)	66 (95.6)	56 (100)	1 (3.6)		0.001
MDR	60 (95.2)	5 (83.3)	65 (94.2)	56 (100)	7 (25)		0.001
Resistance genes, n(%)							
*bla*_OXA-48_	22 (34.9)	4 (66.7)	26 (37.7)	4 (7.1)	0	0.001	0.001
*bla*_NDM_	3	0	3	0	0		
*bla*_TEM_	41 (65)	4 (66.7)	45 (65.2)	45 (80.4)	18 (66.7)		
*bla*_SHV_	29 (46)	2 (33.3)	31 (44.9)	24 (42.1)	5 (18.5)		0.01
*bla*_CTX-M-15_	51 (81)	6 (100)	57 (82.6)	38 (67.9)	20 (71.4)		
CTX-M-G-1	55 (87.3)	6 (100)	61 (88.4)	47 (83.9)	26 (92.9)		
CTX-M-G-2	0	0	0	2 (3.6)	2 (7.1)		
CTX-M-G-8	0	3 (50)	3 (4.3)	0	0		
CTX-M-G-9	20 (31.7)	0	20 (29)	7 (12.5)	13 (46.4)	0.03	
CTX-M-G-25	18 (28.6)	3 (50)	21 (30.4)	5 (8.9)	2 (7.1)	0.004	0.01
*aac6-Ib-cr*	43 (68.3)	2 (33.3)	45 (65.2)	39 (69.6)	7 (25)		0.001
*qnrB*	9 (14.2)	0	9 (13)	8 (14.5)	3 (10.7)		
*qnrS*	6 (9.5)	0	6 (8.7)	11 (19.6)	5 (17.9)		
*ArmA*	4 (6.3)	0	4 (5.8)	6 (10.7)	0		
*aac6-Ib*	42 (66.7)	2 (33.3)	44 (63.8)	36 (64.3)	6 (21.4)		0.001
*aac3-IIa*	40 (63.5)	3 (50)	43 (62.3)	42 (75.1)	9 (32.1)		0.01

### Characterization of resistance genes

The predominant resistance gene detected among the three groups of isolates was CTX-M-G1, followed by *aac6Ib-cr* in ST131 (45, 65.2%), *bla*_TEM-_ in non-ST131 (45, 80.4%) and *bla*_CTX-M-15_ in broiler (20, 71.4%) isolates. The prevalence of *bla*_SHV-_, *bla*_CTX-M-15_, CTX-M-G1, CTX-M-G8, CTX-M-G9, CTX-M-G25 and *bla*_OXA-48_ was higher among ST131 as compared with clinical non-ST131 isolates; however the differences were only significant for the last three resistance determinants. The broiler isolates showed higher prevalence of CTX-M-G1 and CTX-M-G9 than both of two clinical groups (Table 1). The main gene contributing to the ESBL phenotype among the ST131 strains was CTX-M-G1 (60/66, 90.9%), followed by *bla*_CTX-M-15_ (54/66, 81.8%), while in non-ST131 isolates, it was CTX-M-G1 (47/56, 83.9%) and *bla*_TEM-_ (45/56, 80.4%). The *bla*_CTX-M-15_ was more frequently found among ST131 (57/69, 82.6%) than non-ST131 (38/56, 67.9%) and broiler (20/28, 71.4%) isolates, however, the differences were not significant. Among the clinical isolates carrying *bla*_CTX-M-15_, resistance rates were higher against gentamicin (57.9%, *P*: 0.01), amoxicillin-clavulanate (73.7%, *P*, 0.02), cefepime (69.5%, *P*: 0.005), amikacin (15.8%, *P*: 0.02) and tobramycin (65.3%, *P*: 0.001). The *bla*_OXA-48_ and *bla*_OXA-48/NDM_ combination were detected in 23 (33.3%) and three (4.3%) of the ST131 isolates respectively, of those 19 isolates including three *bla*_NDM_ carrying strains were found susceptible to carbapenems. Two out of the four (7.1%) *bla*_OXA-48_ carrying non-ST131 isolates were also categorized as susceptible to carbapenems.

### Phylogrouping

All the 153 strains formed seven distinct phylogroups (A, B1, B2, C, D, E and F). Table 2 shows the phylogroup distribution for the *E. coli* isolates stratified by source and by ciprofloxacin susceptibility pattern. Among the total population, group B2 was the most prevalent phylogroup, corresponding to 55.6% (85/153) of the isolates, followed in prevalence by the other groups: A (17%), E (13.1%), F (5.2%), B1 and C (each 3.9%), and D (1.3%). Almost all ST131 isolates were from group B2 (98.6%, 68/69), whereas the non-ST131 and broiler isolates exhibited diverse phylogroups, with group A being predominant in these two groups. Considering the clinical non-ST131 and broiler isolates, fluoroquinolone resistant strains found mainly belonged to phylogroup A, although the differences were not significant with respect to phylogroup distribution of resistant isolates. None of broiler isolates were identified as ST131 clonal group. Multiplex ST-PCR detected one urine cultured non-ST131 isolate as ST95 (phylogroup A), and broiler isolates did not belong to the investigated five major ST.

**Table 2:** Phylogenetic group distribution for 153 *E. coli* strains stratified by source (human and broilers) and by ciprofloxacin susceptibility status

Phylogenetic group	Ciprofloxacin susceptible strains, *N* = 16			Ciprofloxacin resistant isolates *N* = 137		
	ST131 (*n* = 6) n (%)	Non-ST131 (*n* = 0)	Broilers (*n* = 10) n (%)	ST131 (*n* = 63) n (%)	Non-ST131 (*n* = 56) n (%)	Broilers (*n* = 18) n (%)
A	–	–	2 (20)	–	15 (26.8)	9 (50)
B1	–	–	2 (20)	–	3 (5.4)	1 (5.6)
B2	6 (100)	–	4 (40)	62 (98.4)	13 (23.2)	0
C	–	–	0	–	5 (8.9)	1 (5.6)
D	–	–	1 (10)	–	1 (1.8)	0
E	–	–	0	–	13 (23.2)	7 (38.9)
F	–	–	1 (10)	1 (1.6)	6 (10.7)	0

### Virulence genotyping

**Fig. 1:**
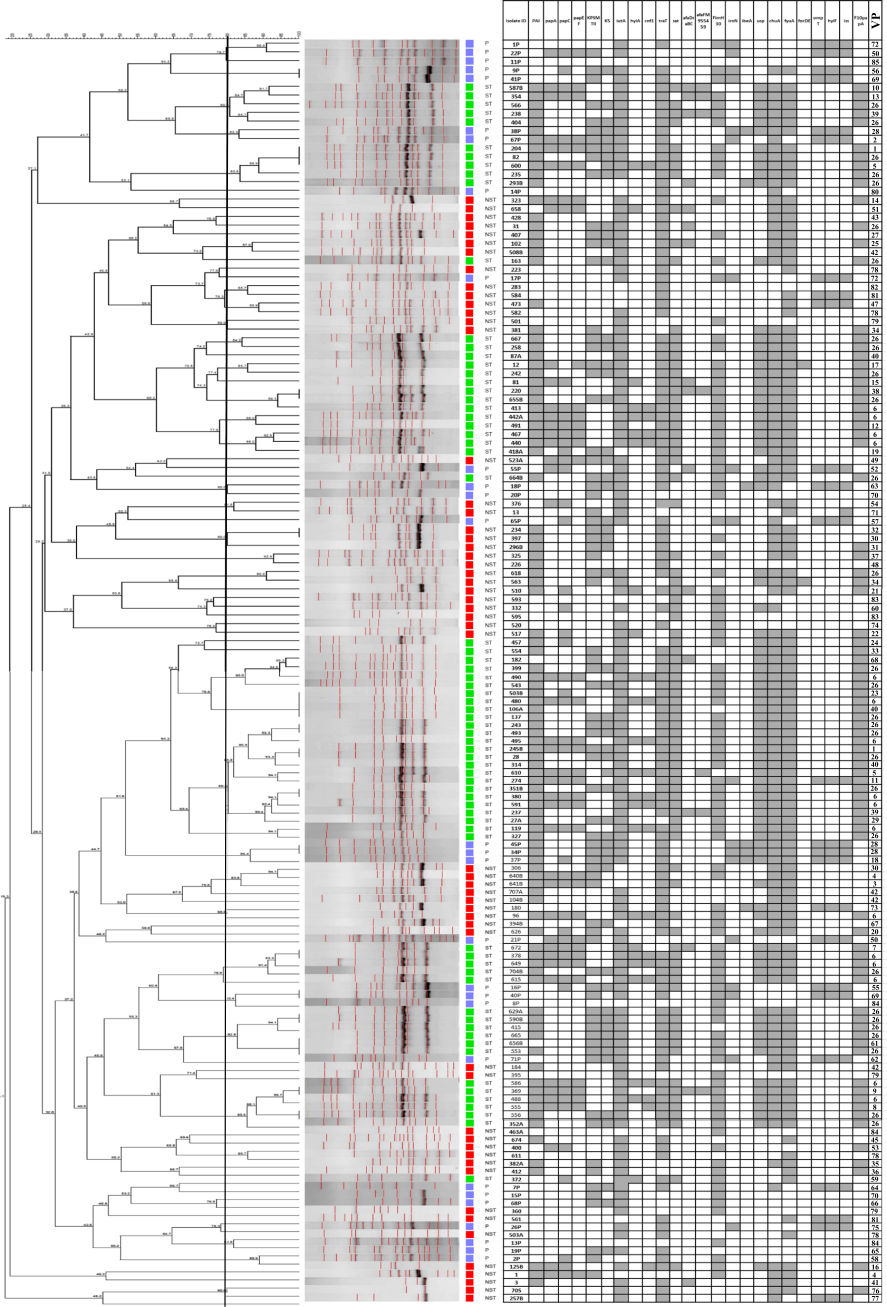
ERIC-PCR based dendogram of the 152 *E.coli* isolates and virulence gene profile. Cluster analysis of the Dice similarity indices based on the unweighted pair group method using average linkages (UPGMA) was done to generate a dendogram describing the relationship among the ERIC profiles. (One non-ST131 isolate didn’t amplify in ERIC-PCR, so it is not included in the dendogram image). P, poultry (broilers); ST, ST131; NST, non-ST131; VP, virulence profile number. Filed squares indicate the presence of the gene

Based on the molecular definition of ExPEC, all ST131 isolates except one O16 and two O25b strains were attributed with the status of ExPEC, in contrast to only 42.9% (24/56, *P*: 0.001) and 75% (21/28, *P*: 0.006) of clinical non-ST131 and broiler isolates, respectively. Considering the 84 non-ST131 isolates, including both of the clinical and broiler strains, fluoroquinolone susceptible isolates showed higher virulence scores as compared with fluoroquinolone resistant strains (median; 11 vs. 6.50, *P*: 0.045). The conserved pattern of virulence genes (i.e., gene prevalence > 90%) of the ST131 isolates included *fyuA, F10 papA*, *iutA*, *chuA*, *sat*, *malX*, *usp* and *traT*. Eighty-five different virulence profiles were found (named 1–85). Twenty of them grouped more than one isolate; profile 26 was the most frequently identified (*n* = 28), while profile 11 showed the highest virulence score (one ST131 isolate, virotype B, virulence score: 16). Clinical non-ST131 and broiler isolates were spread among 42 and 21 different virulence profiles, respectively, whereas ST131 isolates showed 25 different profiles, with the virulence profiles number 26 (*n* = 26) and 6 (*n* = 15) containing the majority of isolates (Fig. 1). A single pattern (pattern 84; *fimH*30^+^) was detected between human (non-ST131) and broiler isolates.

Among ST131 isolates, the prevalence of *K5* and *afaDraBC* was associated with O25b (fluoroquinolone resistant) and O16 (fluoroquinolone susceptible) subgroups, respectively. The presence of *ompT*, *hlyF*, *iss*, *ibeA*, *iroN* and *kpsMT* II genes was associated with broiler isolates. The median virulence score and the virulence score range values decreased progressively from a high of 11 (IQR: 3, range 7–16) for ST131 isolates to a low of 8.50 (IQR: 8, range, 0–14) for broiler isolates, to a score of 6 (IQR: 5, range, 1–14) for non-ST131 isolates (*P* < 0.001) (Table 3).

**Table 3:** Virulence genotypes of studied isolates

ST131 (*n* = 69)				Non-ST131 (*n* = 84)			
Adhesions	O25b-ST131 *n* = 63	O16-ST131 *n* = 6	Total ST131	Human (*n* = 56)	Broiler (*n* = 28)	*P* value	
						*ST131* vs. *non-ST131*	*ST131* vs *avian*
Virulence markers, n (%)							
*papA*	27 (42.8)	1 (16.7)	28 (40.6)	10 (17.9)	4 (14.3)	0.007	0.01
*F10 papA*	61 (96.8)	6 (100)	67 (97.1)	17 (30.4)	0	0.001	0.001
*papEF*	26 (41.3)	1 (16.7)	27 (39.1)	7 (12.5)	3 (10.7)	0.001	0.007
*papC*	30 (47.6)	1 (16.7)	31 (44.9)	14 (25)	9 (32.1)	0.02	–
*sfa/focDE*	1 (1.6)	0	1 (1.4)	1 (1.8)	0	–	–
*afaFM955459*	5 (7.9)	1 (16.7)	6 (8.7)	0	0	0.03	–
*afa/draBC*	7 (11.1)	3 (50)	10 (14.5)	5 (8.9)	1 (3.6)	–	–
*fimH30*	62 (98.4)	0	62 (89.9)	27 (48.2)	17 (60.7)	0.001	0.003
Siderophores							
*fyuA*	63 (100)	6 (100)	69 (100)	44 (78.6)	7 (25)	0.001	0.001
*iroN*	1 (1.6)	0	1 (1.4)	0	17 (60.7)	–	0.001*
*iutA*	62 (98.4)	5 (83.3)	67 (97.1)	39 (69.6)	23 (82.1)	0.001	0.02
*chuA*	62 (98.4)	6 (100)	68 (98.6)	35 (62.5)	12 (43)	0.001	0.001
Toxins							
*cnf1*	18 (28.6)	0	18 (26.1)	2 (3.6)	0	0.001	0.001
*sat*	62 (98.4)	5 (83.3)	67 (97.1)	18 (32.1)	1 (3.6)	0.001	0.001
*hlyA*	20 (31.7)	0	20 (29)	4 (7.1)	0	0.003	0.001
*hlyF*	1 (1.5)	0	1 (1.4)	6 (10.7)	19 (67.9)	0.04	0.001*
Capsules							
*kpsM T II-K5*	34 (54)	0	34 (49.3)	10 (17.9)	13 (46.4)	0.001	–
*kpsMT II-K2*	32 (50.8)	2 (33.3)	34 (49.3)	20 (35.7)	20 (71.4)	–	0.04*
Miscellaneous							
*ibeA*	0	1 (16.7)	1 (1.4)	1 (1.8)	5 (17.9)	–	0.007*
*PAI (malX)*	61 (96.8)	5 (83.3)	66 (95.7)	32 (56.1)	5 (17.9)	0.001	0.001
*usp*	62 (98.4)	6 (100)	68 (98.6)	10 (17.9)	6 (21.4)	0.001	0.001
Protections							
*traT*	60 (95.2)	6 (100)	66 (95.7)	43 (76.8)	20 (71.4)	0.002	0.002
*ompT*	1 (1.5)	0	1 (1.4)	6 (10.7)	19 (67.9)	0.04	0.001*
*iss*	1 (1.5)	0	1 (1.4)	7 (12.5)	18 (64.3)	0.02	0.001*
Virulence score (median)	11	8.50	11	6	8	0.001	0.001
Strains with ≥8 virulence genes	62 (98.4)	6 (100)	68 (98.6)	20 (35.7)	16 (57.1)	0.001	0.001
ExPEC status	62 (98.4)	4 (66.7)	66 (95.6)	24 (42.9)	21 (75)	0.001	0.006

*: indicate the positive association with broiler isolates

Regardless of ST131 status, *H*30 positive isolates were overwhelmingly fluoroquinolone resistant (105/106, 99.1%) (*P*: 0.001). *H*30Rx sublineage was mainly detected among ST131 isolates (27/69, 39.1%), whereas one and four strains of broiler and non-ST131 isolates belonged to *H*30Rx sublineage, respectively (*P*: 0.001). Resistant phenotypes which were associated with *H*30Rx subclone were against cefotaxime, amikacin, tobramycin, gentamycin, fluoroquinolones, amoxicillin-clavulanate and aztreonam. Almost all (31/32, 96.8%) *H*30Rx isolates were qualified as ExPEC and were associated with CTX-M-G1, CTX-M-G9 (*P*: 0.01 for both) and *aac6-Ib*/ *aac6Ib-cr* (*P*: 0.001, for both). *H*30Rx isolates showed higher prevalence of CTX-M-15 gene as compared with *H*30-non-Rx subgroup, but the difference was not significant (87.5% vs. 71.9%). *H*30Rx sublineage of ST131 clone was significantly associated with the virulence genes of *cnf1*, *hlyA*, *k5*, *kpsMT* II, *papA, papC* and *papEF*.

### Genotyping

The highest similarity rates were observed among ST131 isolates, followed by the non-ST131 strains and then the broiler isolates (Fig. 1). Two out the three *bla*_NDM_ carrying isolates exhibited > 80% similarity in ERIC-PCR based fingerprinting. Several dissimilar isolates belonging to all the three groups harboring *bla*_CTX-M-15_ and/or *bla*_OXA-48_ were scattered throughout the dendogram, suggesting possible horizontal gene transfer.

## Discussion

Alarming increase in the isolation of CTX-M-15 producing *E. coli* isolates have been reported from different countries and this phenomenon has been linked to the clonal expansion of ST131 [14, 15]. The association of ST131 with higher ExPEC fraction and multidrug resistance phenotype, together with their ability for widespread dissemination, has led to the worldwide predominance of ST131 among other *E. coli* populations. Past studies have reported the association of selected factors with ST131 and non-ST131 isolates [16]. However, comprehensive analyses to study the association of several determinants with clinical ST131, non-ST131 and *E. coli* isolates from broiler subjects, particularly from developing countries like Iran, remain scarce.

To our knowledge, this is the first study of phenotypic and molecular traits of ST131 and its main subclones, including *H*30 and *H*30Rx and non-*H*30 strains within a population of systematically collected *E. coli* clinical isolates from Iran. We found that among fluoroquinolone resistant isolates, ST131 clone shows priority over non-ST131 isolates including more virulence genes profiles, higher proportion of ExPEC and high similarity for phylogroup B2 background (98.4% vs. 23.2%).

We found a predominance of *bla*_CTX-M-15_ (82.6%) among ESBL producing ST131 *E. coli* isolates. Notably, approximately 60% of non-ST131 and broiler isolates also harbored *bla*_CTX-M-15_, providing evidence of the extended prevalence of *bla*_CTX-M-15_ and a significant association of ST131 with *bla*_CTX-M-15_. Moreover, the *bla*_CTX-M-15_ was significantly associated with resistance to first line antibiotics, notably amoxicillin/clavulanate, aminoglycosides and cefepime, and also among clinical non-ST131 isolates with greater resistance scores. This event would help the rising expansion of *bla*_CTX-M-15_ carrying strains over other ESBL producing *E. coli* isolates in the presence of selection pressure due to the corresponding antibiotics [17].

Studies have reported a core set of virulence factors among ST131 strains including (*fimH*, *fyuA*, *iutA*, *traT*, *malX*), with occasional variation [14, 18]. In our study, these core virulence determinants were detected in > 89% of ST131 isolates screened, along with *F10 papA*, *chuA* and *usp*. Because the present ST131 strains were significantly more likely to be from group B2 than the non-ST131 and broiler *E. coli* isolates, they may have fitness priority over other *E. coli* isolates.

The proportions of *fimH*, *fyuA*, *iutA*, *traT*, and *PAI* genes were similar to those in previous reports [19, 20], although the proportions of *hlyA*/*cnf1* and *ompT/sat* were different from those reported in previous Korean/Indian and U. S studies [20–22]. This result suggests that differences in virulence profiles among ST131 strains may depend on their geographical origin, whether the study was hospital/ “long-term care facility” or community based, and the source of isolates.

The high diversity of virulence gene profiles that we describe in our ESBL producers has been previously described [23], but we found that ST131 has less profile diversity, which implies to the selective distribution of certain sublineages within ST131. Furthermore, the similarity of broiler isolates with human pathogenic *E. coli* based on virulence markers was confirmed in this study, since a total of 75% of broiler isolates belonged to the ExPEC fraction. As also suggested by other studies, certain APEC subgroups, especially a large fraction of phylogroup A isolates may be considered potential zoonotic agents [24].

In this study, evaluation of the potential zoonotic risk of the *E. coli* isolates from broiler relies on the phylogroup distribution associated with ciprofloxacin resistance. Regarding the phylogroups, overall, the proportion of group A strains of clinical non-ST131 isolates was greater than the other phylogroups. Considering that half of the fluoroquinolone resistant broiler *E. coli* isolates we tested belonged to group A and that chicken meat has previously been found prevailed by A and B1 isolates [25], this finding suggests the hypothesis that avian might be a source for at least some human ciprofloxacin resistant group A strains. The change in phylogroup background of isolates from group B2 in drug-resistant ExPEC has been previously observed [26]; however, the appearance and expansion of the ciprofloxacin-resistant B2 ST131 isolates which was also dominant among this clone seems to disagree with these observations [27].

Interestingly, we didn’t find any document indicative for the carriage of ST131 isolates by broiler species which is in agreement with previous reports of rare isolation of ST131 positive cases among avian sources [27]. However, the main limitation of our study was the relatively small number of broiler isolates which restricted statistical power. Based on our results, the ST131 clone is associated with human microbiota and also predominates among fluoroquinolone-resistant strains, while it can be present among susceptible isolates, even in a low number.

## Conclusion

The present study confirms that the *H*30 allele is prevalent among fluoroquinolone resistant isolates. Our data suggest that ST131 clone possess more virulence traits but less multidrug resistance patterns than other clinical fluoroquinolone resistant/ESBL-producing isolates. Among ESBL-positive isolates, ST131 isolates—mostly representing the *H*30-Rx subclone— have higher virulence scores than non-ST131 isolates, implying higher virulence power and thereby the possible reason for their high prevalence. Non-ST131 isolates including both of the clinical and broiler strains showed an inverse relationship between the prevalence of virulence markers and the resistance phenotype in which higher resistance is accompanied by lower frequency of virulence traits. Further work is needed to be done to understand the significance of avian *E. coli* isolates contributing to human disease.

## Methods

### Isolate collection

In this one year cross-sectional study (March 2015–March 2016), a total of 338 *E. coli* isolates were cultured from patients with extra-intestinal infections admitted to Kosar university hospital of Semnan, Iran. The clinical samples were taken as part of standard care for the patients. Sixty-three out of the 338 isolates had been previously described [13]. Moreover, a total of 28 tissue swabs of culled-animals affected with colibacillosis arrived to the laboratory at the same time period. Samples were taken from chickens already sacrificed for diagnostic purposes. The colibacillosis samples were collected from cases submitted to the Diagnostic Service of the Veterinary School of the Semnan University (Semnan Province). Swabs were taken from two broiler farms located in Semnan Province. The samples were plated onto MacConkey agar and incubated overnight at 37 °C [28].

### Antibiotic susceptibility testing and phenotypic detection of ESBL producers

Antibiotic susceptibility patterns for collected *E. coli* isolates were determined by using the standard disc diffusion. The results of disc diffusion were interpreted based on the Clinical Laboratory Standard Institute (CLSI) recommendations [29]. We considered intermediate susceptibility patterns as resistant. Isolates with an inhibition zone of < 23 mm to any of the imipenem, meropenem, or ertapenem were categorized as carbapenem-resistant [29]. The resistance score was the number of antibiotics to which an isolate was resistant. Multidrug-resistant (MDR) was defined according to resistance to at least one representatives of ≥3 antimicrobial classes [30]. Phenotypic combined disc test based on the CLSI recommendations was used to detect the Extended Spectrum β-lactamase (ESBL) producing isolates [29].

### Phylogenetic analysis and sequence typing PCR

The CTAB method was used for extraction of DNA templates for PCR [31]. Major *E. coli* phylogroups (A, B1, B2, C, D, E and F) were determined by quadruplex PCR [32]. For detection of major ExPEC STs (ST131, ST95, ST73, ST69 and ST127), multiplex PCR was used as described previously [33].

### Screening of ST131clonal group

Single nucleotide polymorphism (SNP) in *mdh* and *gyrB* genes associated with ST131 clone was detected by using of the *gyrB/mdh* duplex PCR for all isolates belonged to phylogroups B2, D and F. The *H*30 and *H*30-Rx subclones of ST131 isolates were detected, and the two most common O genotypes, O25b and O16 were screened by PCR among study ST131 isolates [13].

### Virulence genotyping

The presence of 24 putative virulence genes was assessed by multiplex PCR [34]. Isolates which were positive for ≥2 of the five markers, including *afaDrBC*, *papAH*, and/or *papC*, *sfa*, *KpsM II*, or *iutA* were considered as ExPEC, and the virulence score was calculated as described previously [35]. The genes described previously by Johnson et al as the minimal predictors of APEC virulence; *iroN*, *ompT*, *hlyF*, *iutA* and *iss* were detected by a multiplex PCR [36].

### Detection of resistance genes

Isolates carrying resistance markers, including carbapenemases (*bla*_NDM_, *bla*_OXA-48_, *bla*_IMP-_, *bla*_VIM-_ and *bla*_KPC_) [37], (β-lactamases) (*bla*_TEM_-, *bla*_SHV_-,*bla*_CTX-M_ groups 1, 2, 8, 9, 25) [38, 39] and plasmid mediated quinolone resistance (PMQR) (*qnrA*, *qnrB*, *qnrS* and *aac6Ib-cr*) were detected by multiplex PCR according to previously published methods [40]. The presence of the *bla*_CTX-M-15_ was detected using a single PCR [41]. Moreover, aminoglycoside resistance determinants (ARD), including *aac6-Ib*/*aac3-IIa* and 16S *rRNA* methylase genes (*armA*, *rmtB*, *rmtC*) were screened by PCR [42, 43].

### ERIC-PCR analysis

Clonal relatedness was established by Enterobacterial repetitive intergenic consensus sequence polymerase chain reaction (ERIC-PCR). BioNumerics software, version 6.1, (Applied Maths, Sint-Martens-Laten, Belgium) was used to analyz the ERIC patterns. The similarities in amplicon profiles were compared using a Dice coefficient at 1% tolerance and 0.5% optimization, and by using the unweighted-pair group method with arithmetic mean clustering (UPGMA) method, with a cut-off of 80% similarity a dendrogram was constructed [44].

### Statistical analysis

Continuous variables were described as means and standard deviations or as medians and interquartile ranges (IQRs). Dichotomous variables were described using frequencies and percentages, and they were compared using chi-square test, as appropriate. The criterion for statistical significance was *P* < 0.05. Data were analyzed with software SPSS version 16.

## Abbreviations

APEC: Avian pathogenic *E.coli*
*E. coli*: *Escherichia coli*
ECOR: *E. coli* reference collection
ERIC-PCR: Enterobacterial repetitive intergenic consensus sequence polymerase chain reaction
ESBL: Extended Spectrum β-lactamase
ExPEC: Extraintestinal Pathogenic *E.coli*
MDR: Multidrug-resistant
MLST: Multilocus sequence typing
NMEC: Neonatal meningitis *E. coli*
ST: Sequence type
UPEC: Urinary pathogenic *E. coli*
UPGMA: Unweighted-pair group method with arithmetic mean clustering
